# A tissue-specific landscape of sense/antisense transcription in the mouse intestine

**DOI:** 10.1186/1471-2164-12-305

**Published:** 2011-06-10

**Authors:** Ulrich C Klostermeier, Matthias Barann, Michael Wittig, Robert Häsler, Andre Franke, Olga Gavrilova, Benjamin Kreck, Christian Sina, Markus B Schilhabel, Stefan Schreiber, Philip Rosenstiel

**Affiliations:** 1 Institute of Clinical Molecular Biology, Christian-Albrechts-University, Kiel, Germany; 2 Department of General Internal Medicine, Christian-Albrechts-University, Kiel, Germany

## Abstract

**Background:**

The intestinal mucosa is characterized by complex metabolic and immunological processes driven highly dynamic gene expression programs. With the advent of next generation sequencing and its utilization for the analysis of the RNA sequence space, the level of detail on the global architecture of the transcriptome reached a new order of magnitude compared to microarrays.

**Results:**

We report the ultra-deep characterization of the polyadenylated transcriptome in two closely related, yet distinct regions of the mouse intestinal tract (small intestine and colon). We assessed tissue-specific transcriptomal architecture and the presence of novel transcriptionally active regions (nTARs). In the first step, signatures of 20,541 NCBI RefSeq transcripts could be identified in the intestine (74.1% of annotated genes), thereof 16,742 are common in both tissues. Although the majority of reads could be linked to annotated genes, 27,543 nTARs not consistent with current gene annotations in RefSeq or ENSEMBL were identified. By use of a second independent strand-specific RNA-Seq protocol, 20,966 of these nTARs were confirmed, most of them in vicinity of known genes. We further categorized our findings by their relative adjacency to described exonic elements and investigated regional differences of novel transcribed elements in small intestine and colon.

**Conclusions:**

The current study demonstrates the complexity of an archetypal mammalian intestinal mRNA transcriptome in high resolution and identifies novel transcriptionally active regions at strand-specific, single base resolution. Our analysis for the first time shows a strand-specific comparative picture of nTARs in two tissues and represents a resource for further investigating the transcriptional processes that contribute to tissue identity.

## Background

A transcriptome is the complete set of transcripts in a cell, a tissue or a whole organism at a given point in time, and may be altered by developmental stage or environmental stimuli. Transcriptome plasticity is conferred not only by altering the concentration levels of transcripts, but also by complex changes in the architecture of transcripts (splice isoforms, editing, transcription start and termination sites). Measuring the transcriptome is a key point in the decipherment of molecular constituents and in understanding functional elements of the genome, and leads to a better insight into cellular dynamics, for example during development or disease. In the past various technologies have been reported to deduce and quantify the transcriptome, including hybridization- and sequence-based methods. Sequence-based data was intensively used for transcript annotation projects in order to get insight into the complexity of the transcriptome, including expressed sequence tag (EST) projects [1], functional annotation of the mouse (FANTOM) [2-4] and encyclopedia of DNA elements (ENCODE) [5], which represent milestones in our understanding of the transcriptionally landscape in humans and mammalian model organisms.

Emerging next generation sequencing (NGS) technologies allow for an ultra-deep and highly parallel sequencing of complete transcriptomes of individual cells or tissues under study and overcome several limitations of previous technologies [6]. RNA-Seq has been applied to various organisms [7-10] including mouse, highlighting accurate detection of gene expression [11], observation of complex alternative splicing patterns [12,13] and detection of novel transcriptionally active regions (nTARs) in the genome [14]. Although the mouse has already been in the scope of RNA-Seq studies, only a few individual tissues or cell types were analyzed, including embryonic stem cells [15,16], oocytes [17], myoblasts [18], brain [19,20], muscle, liver [11] and heart [21]. Deep annotation of the intestinal mRNA sequence space is still missing, although microarray studies suggested a high complexity of region-specific expression patterns [22,23] and disturbances of intestinal homeostasis are linked to a broad variety of diseases (e.g. infections, idiopathic inflammatory bowel disease and intestinal malignancies) [24,25]. In this study, we introduce a two-step RNA-Seq approach using two different library preparation protocols on the SOLiD platform to characterize the full complexity of an archetypal mammalian intestinal mRNA transcriptome. The method aims specifically to identify novel transcribed elements as well as to describe their orientation in relation to known transcripts.

## Results

### Generation of RNA-Seq data

Total RNA from liquid nitrogen-frozen total small intestine or colon tissue of 9-10 week old C57B6 mice (housed under SPF conditions) was isolated and enriched for polyadenylated RNA approach. Enriched mRNA was reverse transcribed using an oligo-dT nucleotide flanked by a defined sequence and a template switch. Amplification was performed using biotinylated primers to the flanking sequences allowing depletion of the artificially incorporated cDNA ends (Additonal file 1, figure S1). To validate and add strandedness information to our data, another RNA-Seq protocol was used based on direct RNA fragmentation and directional ligation of sequencing adaptors. In total >700 million 35 or 50 bp SOLiD fragment reads were generated from two different tissue sources (colon and small intestine) in a total of six mice. Compared to other recent work the study employs a high total read number as a basis for the analysis [e.g. [11,26]]. Obtained reads were matched to the murine genome (mm9 assembly) using Bioscope software V1.2.1 (Applied Biosystems), only reads mapping uniquely to the genome were further processed for downstream analyses. Mappability of produced reads varied between 48.99% and 66.92%, ~70% of these reads could be mapped to a single position in the genome comparable to other mammalian transcriptome studies [9,19]. A summary containing key data of matching statistics is provided in Table 1.

**Table 1:** Summary mapping statistics small intestine and colon

	cDNA fragmented small intestine	cDNA fragmented colon	RNA fragmented small intestine	RNA fragmented colon
total reads	77,504,263	83,302,412	227,082,832	234,732,979

mapped reads	40,774,275	40,810,609	151,970,014	128,017,728

mapped reads [%]	52.61%	48.99%	66.92%	54.54%

uniquely mapped reads	28,177,719	28,439,065	116,790,095	94,395,393

uniquely mapped reads [%]	69.11%	69.69%	76.85%	73.74%

### Distribution of reads along the 5'-3' axis

**Figure 1:**
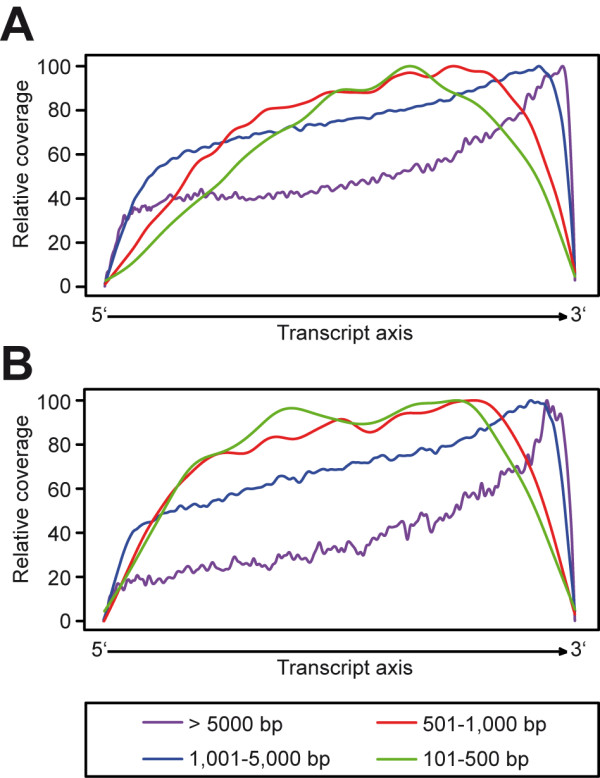
**Transcript length dependent coverage bias along 5'-3' transcript axis**. Transcript length-normalized coverage per base was plotted for several transcript classes. The graph depicts the small intestine data set for oligodT-primed cDNA (SMART protocol) starting from (A) oligodT-purified mRNA and (B) total RNA. Note that especially transcripts > 5000 bp display a stronger bias due to relative overrepresentation of the 3'end.

Oligo-dT primed cDNA generation has been reported to be systematically biased and to preferentially represent 3' ends of transcripts when compared to direct RNA fragmentation [6]. We assumed that this effect is based on incomplete reverse transcription and should be improved by selection for full-length cDNA and template switch. We calculated the genome-wide relative coverage along the 5'-3' axis for different transcript classes sorted by length (Figure 1A). Although the protocol modification led a reduction of the 5´-bias when compared to other studies [7], transcripts of the class of transcripts of more than 5,000 bp length still showed a clear bias towards an overrepresentation of 3' ends, in this class 5' ends showed a relative coverage of ~40%. This effect declines within classes of shorter transcripts. Here a less pronounced depletion of both ends occurred, probably based on primer-containing fragment removal. In another sample (oligo-dT priming without initial purification for polyadenylated mRNA ('total RNA')) this effect was even stronger (Figure 1B). We also investigated the diversity of start points in transcripts with higher abundance and found an interweaving, balanced allocation of reads (exemplified in Additonal file 1, figure S2) in our dataset. No overrepresentation of startpoints with high clonality was observed towards the 5'ends.

### Detection and Quantification of RefSeq transcripts in the intestine

**Figure 2:**
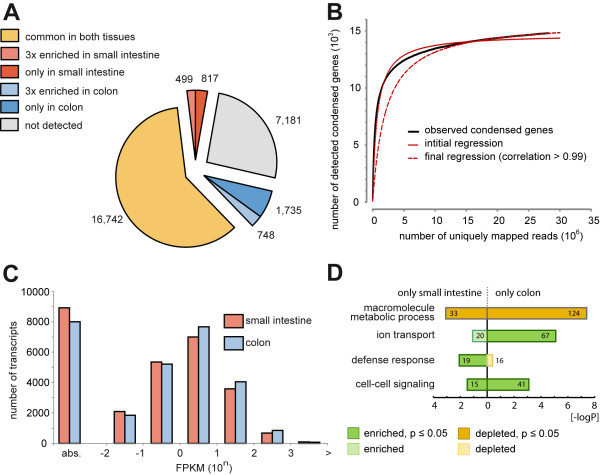
**Detection of RefSeq transcripts in intestinal samples by RNA-Seq**. **(A) **Expression of RefSeq annotated transcripts in the intestine. Number of annotated RefSeq transcripts covered in small intestine and/or colon at a detection threshold of 0.01 FPKM. **(B) **Number of detected condensed genes dependent on sequencing depth (black curve) and graphs depicting regression analysis (red solid and dotted lines). Data depicted from the small intestine data set. **(C) **Distribution of transcripts with different expression levels. All transcripts were sorted to classes spanning an order of magnitude. Transcripts without coverage or not passing the threshold (see (A)) were classified as absent (abs.). **(D) **Enrichment of specific gene ontology terms with key function for the investigated tissues and depletion of 'house keepers' in tissue specific transcript subgroups. Numbers represent the count of transcripts supporting the GO term.

For the estimation of expression levels of RefSeq transcripts (table of February 21^th^, 2011) fragments per kilobase of exon per million fragments mapped (FPKM, [18]) values were calculated using the Cufflinks tool. 20,541 RefSeq transcripts (74.1%) with an expression of more than 0.01 FPKM were considered as present [18,27] in the investigated intestinal tissues. 17,989 transcripts (64,9%) of total RefSeq entries were found to be present in both tissues, 817 (2.95%) were exclusively observed in the small intestine and 1,735 transcripts (6,26%) could be detected only in the colon. Of the shared transcripts, 1,247 transcripts showed a more than 3-fold difference in average coverage (499 enhanced in small intestine vs. 748 in colon) (Figure 2A). To estimate the relative amount of detectable genes to RefSeq annotated genes we performed regression analysis to calculate a saturation curve. RefSeq annotated transcript isoforms were condensed into a single model, including all exons of all isoforms and merging overlapping exons. Of 27,722 transcripts in the RefSeq table 21,923 condensed genes remained. Using a minimum of five reads per condensed gene for detection, 14,801 condensed genes could be identified in the small intestine data set. Plotting the number of detected condensed genes as a function of generated sequencing data clearly shows a saturation kinetic. Data shows that we nearly reached the saturation point for the given library and detection of additional condensed genes would require a huge amount of additional reads. Using a non-linear regression model, we estimated a theoretical maximum of 15,884 condensed genes detectable with at least five reads (Figure 2B).

Many transcripts with highest expression rates in small intestine belong to immune-related processes (e.g. *defensin α6, lysozyme 1*) or nutrient function (e.g. *fatty-acid binding protein 2, cysteine rich protein 1*), in colon most abundant transcripts include effectors of electrolyte transport (e.g. *carboanhydrase 1*) and mucosal protection (*anterior gradient 2, serine protease inhibitor Kazal-type 4*) (a complete list of expression levels is provided in Additional file 2). In total, the detection level of observed transcripts spans several orders of magnitude, a strong fraction of genes show detection levels between 1-10 FPKM (small intestine: 37.20%, colon: 38.87%), about 85% (small intestine: 84.72%, colon 85.83%) of all detected genes showed expression levels between 0.1 FPKM and 100 FPKM (Figure 2C).

For a more general view on differences between the investigated tissues, we performed gene ontology (GO) analysis on subsets of tissue-specific transcripts (i.e. not supported by at least 5 reads and > 2 SPs out of approx. 28 mio. uniquely mapped reads in one of the tissue libraries, but present in the other library). Interestingly, we found a significant enrichment for the GO term cell-cell signaling, ion transport and immune response in both the subsets of colon- and small intestine-specific transcripts, whereas genes supporting the term metabolic processes that would be expected in both tissues were significantly depleted in both samples. Although few of the transcripts underlying each term overlap, the findings strengthen the hypothesis that processes like cell-cell signaling and ion transport are indeed pivotal regulators of tissue identity. Furthermore, the results also strengthen the view that fundamentally different immune processes occur in small and large intestine and may reflect a higher abundance of the MALT in the small intestine. Results of investigated GO terms are listed in Additional file 3. Figure 2D shows enrichment or depletion of mentioned gene ontology terms

### Benchmarking of applied screening protocol and comparison to microarray

**Figure 3:**
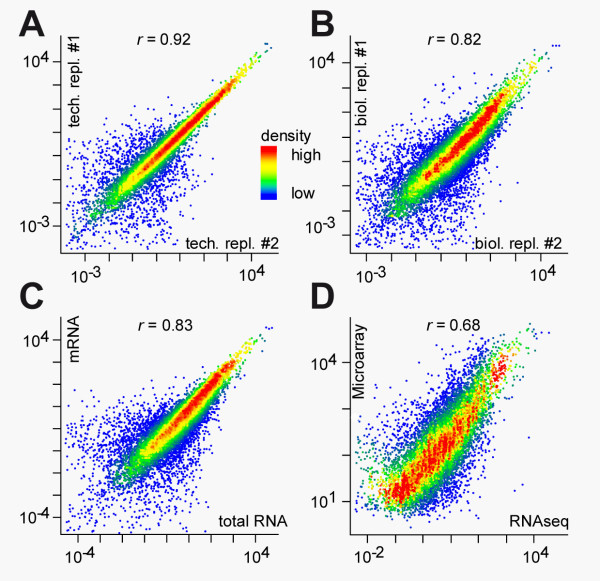
**Reliability of applied method **(A) **The correlation of two technical replicates, **(B) **biological replicates, **(C) **a comparison of either and **(D) **Microarray (y-axis) vs. applied RNA-Seq method (x-axis) is shown and the Spearman rank correlation (r) is stated**.

To investigate the reproducibility of our cDNA sequencing method and to describe interindividual variation of gene expression we performed technical and biological replicates, compared double with single poly-A enrichment (column based poly-A enriched mRNA vs. total RNA as input for oligo-dT primed reverse transcription, which allow input total RNA amounts of less than 1 µg, an overview of sequencing statistics for this samples can be found in Additional file 1, table S1), and correlated our data to the output of standard microarray analysis (Affymetrix Mouse 430 2.0). For our technical replicate, we observed a Spearman rank correlation of 0.92, in the biological replicate using two different individuals from the same animal facility we found a Spearman rank correlation of 0.82. The different protocols for poly-A enrichment showed a Spearman rank correlation of 0.83 (Figure 3 A-C). In comparison with microarray data we found a Spearman rank correlation of 0.68 for transcript levels detectable on both platforms (Figure 3D). As shown for other RNA-Seq protocols our approach is reliable and differences to microarrays are within microarray intra-platform comparisons [28]. High correlation between single and double enrichment of polyadenylated transcripts allow direct use of total RNA with clear reduced input, although the observed depletion of 5' ends along the transcript axis was stronger in the single enrichment experiment.

### Novel identified transcriptionally active regions are clustered in neighbourhood of known genes and orientation is usually in sense orientation to the related transcript

**Figure 4:**
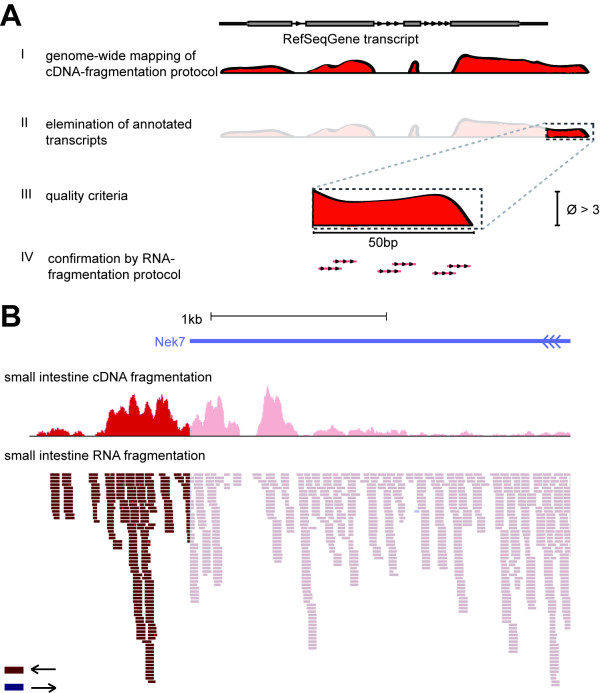
**Strategy for detection and verification of polyadenylated nTARs**. (**A**) All obtained reads from the screening protocol were matched against the mouse genome followed by generation of coverage tracks (I). Covered regions, which are not consistent with either current RefSeqGene or ensGene gene annotation were identified and selected (II). Only nTARs fulfilling our quality criteria (length ≥ 50 bp, average base coverage ≥ 3, III) were further processed and had to be confirmed by a second RNA-Seq protocol with at least 3 reads (IV). (**B**) An example of a nTAR is shown: extension of the last exon of *Nek7 *(*NIMA (never in mitosis gene a)-related kinase 7*), here shown for small intestine (orientation of reads shown by colour, modified screenshot from ucsc genome browser).

**Figure 5:**
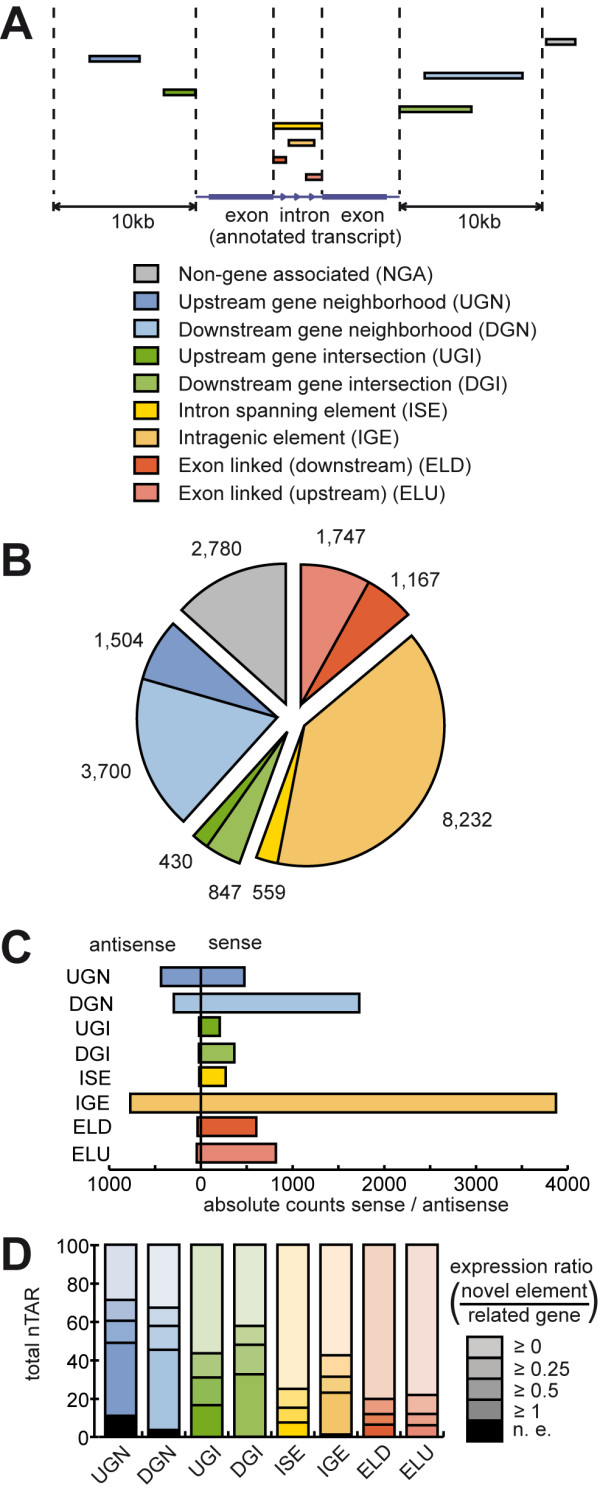
**Characterization of nTARs observed in the murine intestinal transcriptome (A) **Overview of defined classes of novel transcribed elements. Dependent on their relative position to known genes all novel transcribed events were classified. **(B) **Total frequency of novel transcribed elements categorized by the defined classes. **(C) **Pattern of sense / antisense distribution between different classes. Only items of unambiguous orientation were used. **(D) **Comparison of the expression levels of individual novel elements and related genes depicted as expression ratio. Columns show the relative distribution of ratios (n.e.:not expressed).

The salient goal of the present study was the in-depth identification and verification of polyadenylated transcript isoforms, which are unannotated in latest database extracts. For this purpose, the entire sequence space of annotated RefSeq transcripts was deducted from the genome-wide matching. Remaining sequences that mapped uniquely to the genomic backbone were additionally aligned to Ensembl gene annotations (downloaded from UCSC genome browser website [29] on August 29^th^, 2010). Contigs of covered sequence space of at least 50 bp length, which did neither point to RefSeq nor to Ensembl gene annotations, were defined as putative candidates for novel transcripts (nTARs). For the validation of these findings other methods like qPCR or Sanger EST sequencing cannot be realized on a genome-wide level. Thus we chose a different RNA sequencing protocol with significant changes in library preparation. While the first protocol is based on oligo-dT mediated, full length cDNA reverse transcription, template switch and double-stranded cDNA fragmentation, in the second protocol mRNA fragmentation is followed by random hexamer priming. In addition, this protocol added information about the orientation of the investigated nTARs (Figure 4). Mapping statistics showed a slightly higher rate of total and unique mapped reads, possibly due to a longer read length of 50 bp in the SOLiD V4 protocol. Confirmed nTARs were sorted into 9 groups: non-gene associated (NGA) events, which are in more than 10 kb distance to the next annotated transcript. Upstream gene neighbourhood (UGN) and downstream gene neighbourhood (DGN) events are within 10 kb to an annotated gene, but do not overlap. Up- and downstream gene intersecting events (UGI/DGI) are directly connected with the 5'- or 3'-UTR of an annotated gene, while other exon-linked nTARs were classified as exon-linked downstream (ELD) or exon-linked upstream (ELU) events. nTARs, which span a whole intron, are defined as intron-spanning element (ISE), while only partially covered regions of an intron with no overlapping to known exons are described as intragenic element (IGE) (Figure 5A). Using this strategy we could verify 20,966 nTARs of initially 27,543 events observed with the first protocol (76.12%), (Figure 5B for a detailed class report, a list of validated nTARs is contributed in Additional file 4), most of them in close relation to known genes. In proximity (<10 kb) of genes orientation of unambiguous nTARs was usually in sense orientation to neighbouring transcripts with ratios between 5.0 (IGE) and 18.6 (ELU). Only transcribed elements upstream of known transcripts (UGN) displayed a more or less random distribution (ratio 1.1). Despite the predominance of sense events, a vast number of new antisense events could be identified, especially in IGE (n = 769), which could represent regulatory modulators [30] of surrounding genes or even independent transcriptional units (absolute values for each class are shown in Figure 5C, nTARs showing both sense and antisense reads were not considered for sense/antisense pattern of nTARs).

### Basic expression of nTARs is reduced compared to related genes, but can differ between intestinal tissues

**Figure 6:**
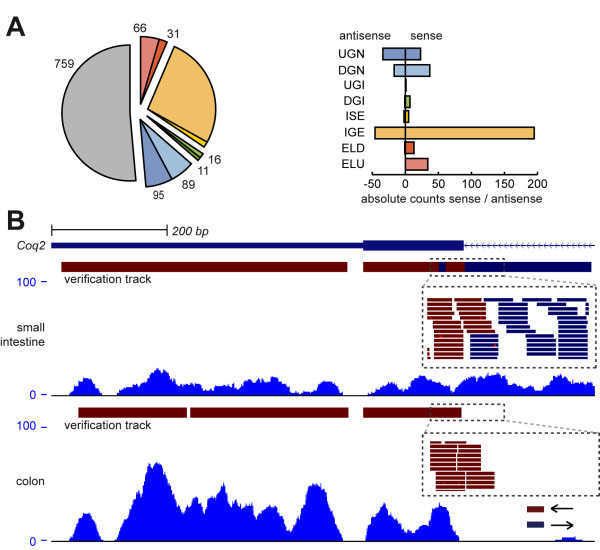
**Differential expression levels of nTARs in investigated tissues**. **(A) **Total counts of nTARs with 3-fold or higher changes in detection levels between tissues. For nTARs joint to a known gene, expression was corrected by fold change of the related gene. While NGA show a high variability, related genes usually are also linked in expression levels of neighbouring genes (for colour code, see Figure 4A). Additionally, orientation of differentially expressed nTARs is shown, compared to the orientation of total counts (Figure 5C), sense orientated findings show reduced expression variability, especially in DGN. **(B) **Example for a differentially expressed nTAR. In the colon, strong expression of *Coq2 *(*coenzyme Q2 homolog, prenyltransferase*) can be observed, but only slight expression in the ELU region of the last exon. In contrast, in the small intestine we observed a lower level of transcript expression, yet the nTAR region upstream of the last exon (ELU event) is distinctly upregulated in comparison to colon samples. Investigating the orientation of involved reads (dashed box) suggests, that this ELU event is transcribed in antisense orientation to the related gene.

To assess the question, how the expression of a given nTAR varies regarding to the related gene, we determined the ratio of the expression levels of a given nTAR and the related neighbouring transcript. 408 nTARs were related to a gene without expression (2.24%). 9,698 nTARs (53.33%) showed a detection ratio (coverage nTAR to coverage related gene) of 0.25 or below showing that the majority is rarely expressed compared to the related gene. This could explain why these genomic regions have not been annotated before. However, the distribution between different classes of nTARs vary significantly (Figure 5D). While intron spanning elements and both exon-linked classes play only minor roles (25% or less show detection ratios of 0.25 or higher), which may point to premature transcripts and/or failed splicing assembly, for nTARs neighbouring the untranslated regions of known genes up to 63.59% (DGN) showed detection ratios of 0.25 or higher. IGE show an intermediate level of well-detected transcripts (41.20%), in contrast to ISE non-mature RNA does not explain the detection of only parts of the intronic sequence. Together with the finding of a quite high rate of antisense reads, this suggests hidden transcriptionally active elements in intronic sequences, which may be additional exons, but also regulatory elements or completely independent transcripts. To further investigate, if detection level of nTARs alters in our two tissue subsets, we calculated the alteration rate between tissues, in case of gene-related nTARs corrected for gene expression changes (Figure 6A). While many NGA show a more than 3x change of expression (27,30%), only 3.98% of gene related nTARs pass this threshold, in particular nTARs at 3' ends of known genes does not change expression compared to the related gene (DGI: 1.89%, DGN: 2.41%). An example of a tissue-specific expressed nTAR is shown in Figure 6B. A complete list of differentially expressed nTARs can be found in Additional file 5. To further corroborate polyadenylation sites, an additional protocol was used based on 3'-anchored pyrosequencing on a 454/GS-FLX [31]. Whereas the coverage depth of pyrosequencing reads was not comparable to the SOLiD data sets (SMART: ~1.8Gb, WTAK: ~12Gb, FLX: < 4 MB unique matchable reads), we could verify 489 of 20,699 novel transcribed elements by at least a single specific 3'-anchored read (Additional file 1, figure S3, Additional file 6). The majority of 3'-anchored ends of nTARs related to a gene was confirmed in sense direction to their transcripts downstream of the reported transcript end. High rates of sense nTARs point together with these findings of a direct incorporation into known transcripts for many findings, especially for elements at the 3' UTR of known genes. However, within nTAR classes UGN and IGE we could observe also a high rate of polyadenylation signals in antisense direction to the known genes.

## Discussion

Our findings describe a novel two-step RNA-Seq approach to systematically identify novel transcribed elements and for the first time present a view on the landscape of gene expression of the murine intestinal tract by means of massively parallel sequencing. Using this method we demonstrate high intestinal transcriptome complexity with expression of 74.1% of RefSeq annotated transcripts. The observed values for uniquely mappable reads are similar to other RNA-Seq studies employing murine and human complex tissues [9,19] Compared to other tissues like the brain investigated by massively parallel sequencing (58.7% of known genes were reported as expressed in embryonic and neonatal mouse brain [19]), the intestine thus shows a higher complexity at a molecular level and the majority of genes are present in both small intestine and colon. RNA-Seq shows almost no background noise and allows an absolute quantification of transcripts [11]. Thus, it is of note that our study clearly demonstrates the tissue-specific absence of certain transcripts, which are covered not even by a single sequence in the small intestine, but present at a relevant per base coverage in the colon (e.g. H^+^/K^+^-Transporter *Atp12a*) or vice versa are only present in the small intestine (e.g. type 2 glucose transporter *SLC2A2*). Highly abundant transcripts observed in our data sets relate to earlier microarray studies, several of these transcripts have been shown to be strongly expressed in the intestine [22]. Several of the most abundant transcripts are well known players in intestinal physiology, e.g. *carbonic anhydrase 1 *in the colon or *fatty acid binding protein 2 *in small intestine tissue. In addition, for exclusively expressed transcripts highest significance values for the enrichment of certain gene ontology terms were found in processes clearly associated with the investigated tissue (e.g. ion transport, cell-cell signaling).

As the detection and quantification of transcripts presented here is based only on a limited number of datasets from two different tissues of the same individuals, conclusions about transcripts as being present in either only colon or only small intestine remain clearly descriptive. Transcripts displaying strong differential expression between colon and small intestine, should be considered only as exemplary observations and may indicate biological processes that are more prominent in one tissue over the other. Using the data as a first blueprint, it will be interesting to discriminate the roles of absent gene expression and rare transcripts in the determination of intestinal tissue identity and function.

Yet, several advantages can clearly be identified in this benchmarking study: (a) Digital gene expression analysis by RNA-Seq has a wide (and to experimental requirements adaptable [32]) dynamic range and also allows a detailed picture of extremely rare transcript forms. (b) Unlike microarrays RNA-Seq is not limited to the detection of *a priori *determined sequences and thus allows the detection of unknown transcripts. We have chosen a two-step approach to identify and validate novel transcribed elements that result in polyadenylated transcripts using two independent RNA-Seq library preparation methods. For the intestine we show a high number of non-annotated regions of transcriptional activity, 20,699 of these could be verified by an independent protocol emphasizing the still limited knowledge on tissue-specific mammalian transcriptome signatures. Interestingly the classification of nTARs in relation to annotated transcripts confirmed a strong clustering in the vicinity of known gene as recently reported for other nTARs in different tissues from mouse [16] and human cell lines [33]. Even though there is evidence for still unknown transcripts expressed in the intestine (NGA), the more considerable lack of information seems to be in the fine structure of known gene loci. (c) The method allows for a simple discrimination and annotation of read strandedness and thus allows for a deeper insight into identified transcriptionally active regions. As example we have focused on the sense-antisense distribution of novel RNA sequences in the vicinity of known transcripts. The majority of identified gene-associated nTARs are in sense orientation, although a distinct number of pure antisense elements and also mixed nTARs could be identified. Most of intronic nTARs are expressed at a lower level when compared to adjacent or directly linked genes. Thus, some of the detected nTARs may also display premature, non-spliced RNA molecules. However, it is plausible that the many of the transcriptionally active regions in the vicinity of known genes are representing tissue-specific modulatory events. In particular, we demonstrate an unprecedented diversity of nTARs at the 5'or 3' border of known genes, which are realized both in sense and antisense direction. While some of the antisense findings may point to novel regulatory antisense transcripts [34], the finding of sense nTARs downstream of known genes highlight the leakiness of many of the known polyadenylation signals [35,36] and point to a highly diverse and tissue-specific realization of 3'-untranslated regions.

### Conclusion

In summary, the current study provides a public data resource for other researchers (e.g. for the identification of context-dependent transcript isoform and/or regulatory antisense transcript expression) and demonstrates the power of RNA-Seq approaches in order to identify novel strand-specific transcriptional units. Our observations may point to complex and so far undetected sense/antisense regulation events in many of the transcripts that warrant functional in-depth investigation and may ultimately lead to novel insights into intestinal biology.

### Materials and methods

#### RNA-Isolation

Total RNA was isolated from liquid nitrogen frozen intestinal tissues of in total 6 9-10-weeks old C57B6 mice (housed under SPF conditions) either using RNeasy mini kit (Qiagen) followed by mRNA enrichment with Oligotex mRNA purification kit (Qiagen) for SMART sequencing or mirVana miRNA isolation kit (Ambion) for use with whole transcriptome analysis kit (WTAK, Ambion). RNA was isolated from either total small intestine tissue (jejunum) or colon tissue (distal colon).

#### Animals

Mice were maintained in a 12-h light-dark cycle under standard conditions and were provided with food and water *ad libitum*. Procedures involving animal care were conducted in conform to national and international laws and policies.

#### RNA-Seq

500 ng enriched mRNA was used for SMART cDNA synthesis. For second strand synthesis and amplification, a 5'-biotinylated version of PCR primer II was employed. 13 cycles of amplification led to a yield of more than 2 µg cDNA. Subsequently, SOLiD V2 fragment library protocol (Applied Biosystems) was applied and transcript ends were depleted by two rounds of Dynabeads M-280 streptavidin (Invitrogen) treatment. For SOLiD WTAK (RNA fragmentation protocol) 10 µg total RNA was enriched for polyadenylated RNA and used as input for library construction following manufacturer's instructions (Applied Biosystems). First type of libraries (cDNA fragmented) was sequenced on a SOLiD V2 and V2.5 (replicates) sequencing by ligation sequencer following manufacturer's instructions, second type of libraries (RNA fragmented) on a SOLiD V4. The full datasets have been submitted to a public data repository (Gene Expression Omnibus, http://www.ncbi.nlm.nih.gov/geo accession number: GSE21746).

#### Mapping algorithm

Colour space reads (.csfasta) were mapped against the mouse genome reference (mm9). For matching SOLiD™ BioScope™ Software V1.2.1 (Applied Biosystems) was employed using a mismatch penalty of -2, i.e. the mapping pipeline first searches for short matches between a read and the reference. For this initial seed we used 30 bp for the 35 bp reads, allowing for up to 3 mismatches and a 38 bp seed for 50 bp reads with up to 3 mismatches. Additionally we used a repetitive mapping scheme for the 50 bp reads, with a 25 bp seed and up to 2 mismatches. Successfully placed seeds are then extended, adding +1 to the score for every match and using a penalty of 2 for each mismatch. Finally the shortest of best scored alignments is chosen, for details see Bioscope user manual at: http://www3.appliedbiosystems.com. The coverage custom tracks represent the visualization of the SAMtools pileup output [37].

#### Oligonucleotide DNA microarray hybridization

Total RNA was processed as previously described [38] and hybridized to an Affymetrix Mouse 430 2.0 array (Affymetrix Inc, Santa Clara, CA) according to the manufacturer's protocol. Data was normalized using RMA (AGCC, Affymetrix) and signals with a detection p-value of = 0.05 were considered as present. Experimental and analytical part of the microarray analysis was performed following the MIAME standards. The datasets have been submitted to a public data repository (Gene Expression Omnibus, http://www.ncbi.nlm.nih.gov/geo accession number: GSE21746).

#### Gene expression

Transcript expression rates were calculated using the bioscope *.bam output files and transcript annotations from the UCSC homepage (refGene table of build mm9, February, 21th 2011). To interrogate expression levels we calculated FPKM using the published tool Cufflinks. As we rely on a small sample set we defined a conservative value of 3-fold difference between the two tissues in order to filter for potentially interesting results. A present/absent threshold was set to 0.01 FPKM as reported previously [18]. Present transcripts were required to have at least two independent start points. Gene Ontology analysis was performed as previously published [39] by comparing genes present or absent only in either colon or small intestine. Biological processes associated to the transcripts were retrieved from the Gene Ontology Consortium (http://www.geneontology.org).

#### Gene saturation plot and estimation of total detectable RefSeq transcripts by regression analyses

To estimate the number of detectable genes, partial included data on transcript isoforms was removed from the RefSeq table by allowing gene symbols only once within the RefSeq table. Transcripts sharing a gene symbol were artificially fused to a 'supertranscript', so that expression of either isoform led to detection of the corresponding gene symbol. Reads were randomly drawn and removed from the entirety of matched reads. Drawn reads located within annotated genes increase the read count of the corresponding transcript by 1. Genes were considered as being present with at least 5 reads in relation to the number of drawn reads. The collected data was used to calculate a non-linear regression based on the following formula:

To further improve the estimation of total expressed genes, the second intersection of this initial regression curve with the experimental collected data points has been determined and for the points on the right side of the intersection another non-linear regression curve has been calculated. This has been repeated until the correlation of the regression curve reached 0.99 and no further improvement could be achieved.

#### Detection of tissue-specific increased gene expression

Enhanced gene expression was defined as a 3-fold increase in FPKM in one tissue compared to the second. To reduce the impact of insubstantial changes of rare expressed transcripts, the FPKM of any transcript in the second tissue was increased by 1 (see equation below). Both tissues were compared to the other one and only transcripts with a 3-fold higher coverage despite penalty were considered as tissue-specific increased.

#### nTAR detection and classification algorithm

To investigate hitherto unannotated but transcriptionally active regions (nTARs) the *.bam files were screened for chained, covered bases which were not present in investigated databases (refGene, ensGene). In order to use a conservative strategy and to avoid a high false positive rate (e.g. around exon/intron boundaries) we chose minimum nTAR length of 50 bp and defined a detection threshold of 5 reads and two independent start points. Although deeper annotations (e.g. FANTOM, ENCODE) exist, we have chosen a design similar to a previous study [33] using a combination of RefSeq and ENSEMBL as standard gene databases to detect novel elements. Depending on the position of the nTAR relative to annotated genes 9 classes were defined. nTARs with a distance to annotated genes greater than 10 kb were classified as non-gene associated (NGA) events. nTARs within the 10 kb range which did not start or end right beside to annotated genes were classified as upstream or downstream gene neighbourhood (UGN, DGN). nTARs starting or ending right beside to annotated genes were classified as upstream or downstream gene intersections (UGI, DGI). All other nTARs were located within annotated genes. nTARs extending exons were classified as exon-linked up- or downstream (ELU, ELD) events. Intragenic elements (IGE) were defined by no overlap with exons, whereas intron spanning elements (ISE) covered a whole intron. Hits fitting into several classes were counted only once following a priority list: ISE, ELD/ELU, UGI/DGI, IGE, UGN/DGN, NGA. Hits in neighbourhood of two genes were assigned to the closest one. To verify the nTARs, reads derived from the RNA fragmentation protocol were investigated. Validation of an nTAR required at least 3 reads from the RNA fragmentation protocol. For all nTARs, RNA fragmentation protocol reads were counted separately for sense and antisense direction (compared to the respective gene). Method and equations for calculation of the detection ratio of nTARs to related genes and differential regulation of nTARs in different tissues can be found in SI methods.

## Authors' contributions

UCK, MB, MW, BK and PR created and expanded the screening strategy and data analysis pipeline. RH generated and analysed microarray data and performed GO analyses. OG and CS contributed to sample generation. UCK and MBS constructed libraries and performed sequencing. SS, AF, UCK and PR elaborated experimental design. The manuscript was prepared by UCK, MB and PR, with the participation of AF and SS. PR and SS supervised the work. All authors read and approved the manuscript.

## Supplementary Material

Additional file 1 **Supplementary information**. Additional figures for workflow of cDNA fragmentation library protocol, distribution of unique start points of a exemplary gene, and polyadenylated nTARs confirmed by GS-FLX Pyrosequencing. Click here for file

Additional file 2 **Intestinal gene expression**. Gene expression values were calculated using Cufflinks for both investigated intestinal tissues. Expression is measured in Fragments Per Kilobase of transcript per Million mapped reads (FPKM). Click here for file

Additional file 3 **Gene ontology analysis**. Gene ontology analysis to investigate the presents or absence of gene ontology terms in either or both tissues. Click here for file

Additional file 4 **List of nTARs**. All novel transcriptionally active regions with a minimum length of 50 bp and support of at least three reads of the second protocol. Click here for file

Additional file 5 **Differentially expressed nTARs**. All nTARs differentially expressed in the small intestine compared to the colon. Click here for file

Additional file 6 **nTARs with poly-A tag**. All nTARs associated with poly-A tags which have been identified by pyrosequencing. In addition, authors provide a browsable transcriptome/genome viewer for easy data examination on their institutional homepage: http://ucsc.ikmb.uni-kiel.de/cgi-bin/hgTracks Click here for file
