# Supplementary material for: A tissue-specific landscape of sense/antisense transcription in the mouse intestine
Source: BMC Genomics. 2011 Jun 10;12:305. doi: 10.1186/1471-2164-12-305 (PMC3125268; doi:10.1186/1471-2164-12-305)
Supplement: Additional file 1 — Supplementary information. Additional figures for workflow of cDNA fragmentation library protocol, distribution of unique start points of a exemplary gene, and polyadenylated nTARs confirmed by GS-FLX Pyrosequencing. [file 1471-2164-12-305-S1.PDF]

SUPPLEMENTARY MATERIAL FOR:

A tissue-specific landscape of sense/antisense transcription in the mouse intestine

Ulrich C. Klostermeier[1], Matthias Barann[1], Michael Wittig[1], Robert Häsler[1], Andre Franke[1], Olga Gavrilova[1], Benjamin Kreck[1], Christian Sina[1,2], Markus B. Schilhabel[1], Stefan Schreiber[1,2] and Philip Rosenstiel[1]

[1] Institute of Clinical Molecular Biology, Christian-Albrechts-University, 24105 Kiel, Germany

[2] Department of General Internal Medicine, Christian-Albrechts-University, 24105 Kiel

[1] To whom correspondence may be addressed:
Philip Rosenstiel (p.rosenstiel@mucosa.de)
Stefan Schreiber (s.schreiber@mucosa.de)
Institute of Clinical Molecular Biology
Christian-Albrechts-University Kiel
Schittenhelmstr. 12
D-24105 Kiel, Germany
Tel.: +49-431-597-2350
Fax.: +49-431-597-1842

SI Methods

nTAR expression compared to related gene. For the calculation of the expression ratio between nTARs and their related gene, the average base coverage was calculated and normalized to the genome wide total coverage (average base coverage per gigabase sequence space, $abc_{(Gb)}$)for any nTARs from the combined tissue data set and divided by the $abc_{(Gb)}$ of the related gene. The expression ratio for nTARs with related genes showing no expression in the investigated tissues, were set to n.e. (not expressed).

$$(ratio) = \frac{(abc_{(Gb)}nTar\ a + \ abc_{(Gb)}nTar\ b)}{(abc_{(Gb)}Transcript_{tissue\ a} + \ abc_{(Gb)}Transcript_{tissue\ b})}$$

Detection of tissue-specific differential expressed nTARs. Differential expression of NGA was calculated as ratio between abc(Gb) of both tissues, to avoid differential expressed nTAR as consequence of insubstantial alterations of ultra-rare expressed transcripts, a penalty of 1 was added to the divisor. For nTARs related to an annotated gene, the tissue fold change was corrected by the fold change of the related gene, insubstantial fold changes again were corrected by adding a penalty of 1 to the divisor:

$$(fold\ change)\ = \frac{[abc_{Gb}nTAR\ a][abc_{(Gb)}transcript\ b]}{([abc_{Gb}nTAR\ b] + 1)\ ([abc_{Gb}transcript\ a] + 1)}$$

Each tissue was compared to the other on, only nTARs with a 3-fold or higher increase in one of the tissues were considered as differential expressed.

Supplementary figures and tables:

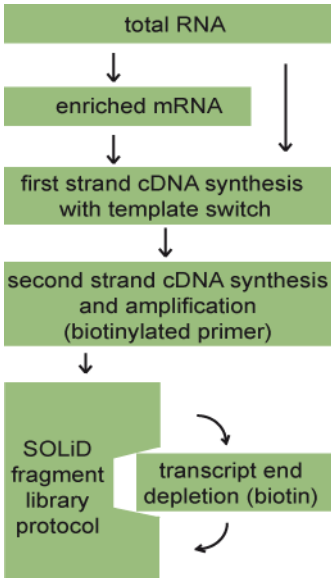

Fig. S1. Schematic overview of cDNA fragmentation library protocol.

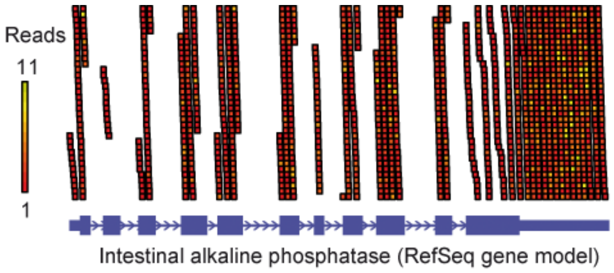

Fig. S2. Intestinal alkaline phosphatase is given as an example for the distribution of unique start points in the sequencing library from small intestine. Each small rectangle represents a unique start point. The number of reads with identical start points is indicated by color.

Tab. S1. Replicate mapping statistics

	technical replicate 1 (B1)	technical replicate 2 (B2)	total RNA input (C)	mRNA input / biological replicate (D)
total reads	25,290,820	34,324,215	27,029,531	24,535,567
mapped reads	13161551	16478454	12891064	12902680
mapped reads [%]	52.04%	48.01%	47.69%	52.59%
uniquely mapped reads	9,040,655	11,341,373	9,004,107	9,370,788
uniquely mapped reads [%]	68.69%	68.83%	69.85%	72.63%

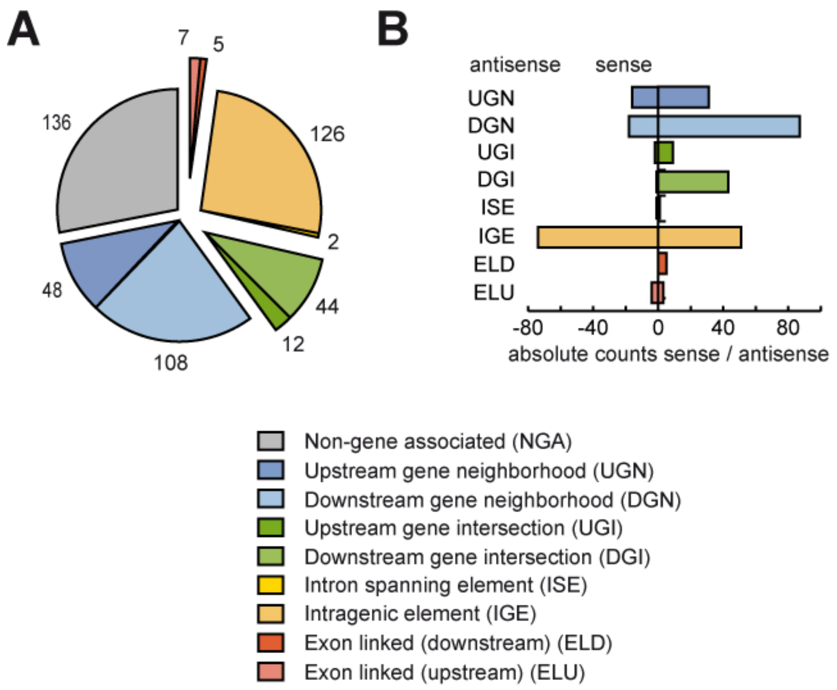

Fig. S3. Polyadenylated 3' ends.

(A) nTARs confirmed by GS-FLX Pyrosequencing derived 3'-poly-A anchored reads are shown. Although coverage depth of SOLiD sequencing was not achieved by pyrosequencing, this approach verifies at least a subset of polyadenylated nTARs and allows insight, in which defined nTAR-classes polyadenylation signals occur. IGE, DGI and DGN show most frequent polyadenylated nTARs, in particular DGI show a disproportional high number of polyadenylated nTARs compared to the total number of detected nTARs. (B) Read orientation of polyadenylated fragments. DGI with a polyadenylated end share usually the orientation of the related transcript. Interestingly, IGE show a more or less random distribution, showing that within this fraction beneath potentially alternative exons with early polyadenylation signals structurally independent transcripts exits.
